# Prostatic lineage differentiation from human embryonic stem cells through inducible expression of NKX3-1

**DOI:** 10.1186/s13287-024-03886-y

**Published:** 2024-09-02

**Authors:** Songwei Wang, Yangyang Yu, Yinglei Li, Tianzhe Zhang, Wei Jiang, Xinghuan Wang, Ran Liu

**Affiliations:** 1 Department of Urology, Department of Biological Repositories, Frontier Science Center for Immunology and Metabolism, Medical Research Institute, Zhongnan Hospital of Wuhan University, Wuhan University, Wuhan, 430071 China; 2 Hubei Provincial Key Laboratory of Developmentally Originated Disease, Wuhan, 430071 China

**Keywords:** Prostatic differentiation, Human embryonic stem cell, Organoid, NKX3-1

## Abstract

**Background:**

Understanding the lineage differentiation of human prostate not only is crucial for basic research on human developmental biology but also significantly contributes to the management of prostate-related disorders. Current knowledge mainly relies on studies on rodent models, lacking human-derived alternatives despite clinical samples may provide a snapshot at certain stage. Human embryonic stem cells can generate all the embryonic lineages including the prostate, and indeed a few studies demonstrate such possibility based on co-culture or co-transplantation with urogenital mesenchyme into mouse renal capsule.

**Methods:**

To establish a stepwise protocol to obtain prostatic organoids in vitro from human embryonic stem cells, we apply chemicals and growth factors by mimicking the regulation network of transcription factors and signal transduction pathways, and construct cell lines carrying an inducible *NKX3-1* expressing cassette, together with three-dimensional culture system. Unpaired t test was applied for statistical analyses.

**Results:**

We first successfully generate the definitive endoderm, hindgut, and urogenital sinus cells. The embryonic stem cell-derived urogenital sinus cells express prostatic key transcription factors AR and FOXA1, but fail to express NKX3-1. Therefore, we construct *NKX3-1*-inducible cell line by homologous recombination, which is eventually able to yield AR, FOXA1, and NKX3-1 triple-positive urogenital prostatic lineage cells through stepwise differentiation. Finally, combined with 3D culture we successfully derive prostate-like organoids with certain structures and prostatic cell populations.

**Conclusions:**

This study reveals the crucial role of NKX3-1 in prostatic differentiation and offers the inducible *NKX3-1* cell line, as well as provides a stepwise differentiation protocol to generate human prostate-like organoids, which should facilitate the studies on prostate development and disease pathogenesis.

**Supplementary Information:**

The online version contains supplementary material available at 10.1186/s13287-024-03886-y.

## Introduction

The prostate is a crucial organ unique to males, positioned inferior to the bladder, approximately the size of a walnut, composed of ducts with an inner layer of epithelium surrounded by stroma. Its primary functions include controlling urination and secreting prostatic fluid, which are of paramount importance to reproductive health [1]. The prostate is derived from the urogenital sinus (UGS) through a series of events including pre-budding, budding, bud elongation and branching, canalization of the solid epithelial cords, and luminal and basal epithelial cell differentiation [1]. The UGS comprises the urogenital sinus epithelium (UGE) and the urogenital sinus mesenchyme (UGM). The nascent prostate is composed of solid epithelial cords in the initial phase of branching morphogenesis. Subsequently, these epithelial cords undergo canalicular differentiation to form glandular structures, with epithelial cells differentiating into basal, luminal, and neuroendocrine lineages, ultimately leading to the formation of a complete prostatic structure [2]. Through the analysis of cellular lineage, it is evident that the entire length of the primitive urogenital sinus from which the prostate originates, is derived from the endoderm [3].

The prostate forms through epithelial budding from the UGS which is initiated by the action of androgens: during the 9–10 weeks of human embryonic development, Leydig cell-secreted dihydrotestosterone (DHT) binds to androgen receptors (AR) and then enters the nucleus to bind to androgen response elements (ARE) in chromatin, thereby activating AR downstream signals in the UGM [4]. Subsequently, mesenchymal AR activates a paracrine signal that acts on UGE to stimulate the formation of buds [5]. As a pivotal transcription factor in endodermal cells, FOXA1 functions as a pioneer factor and enhances the interaction with chromatin, thereby facilitating the binding of AR to the corresponding androgen receptor response elements and promoting transcription efficiency [6]. The high expression level of FOXA1 enhances the AR binding to genomic regions [7, 8]. NKX3-1 is expressed during following prostate differentiation, predominantly in an androgen specific manner [9]. NKX3-1 is considered as a specific marker of the urogenital sinus and is involved in the development of the testis and prostate [10]. The absence of NKX3-1 expression leads to defects in the secretion of prostate proteins and ductal morphology, as well as the appearance of heteromorphic hyperplasia in the urogenital sinus, which can subsequently progress to cancerization [10, 11]. As the core transcription factors of the prostatic fate determination, AR, FOXA1 and NKX3-1 play distinct roles, collectively orchestrating the genesis of the prostate.

Besides of the transcription factors, prostate development is also associated with the modulation of signal pathways. WNT pathway can regulate the morphology and genesis of the urogenital sinus branches as well as the differentiation and proliferation of luminal epithelial cells. A local upregulation of WNT ligands is observed during the formation and budding of prostate branches [12, 13], and WNT inhibitors significantly reduce the budding of the prostate and suppress the expression of NKX3-1 as well as the differentiation of luminal epithelial cells [14], together indicating that the WNT pathway plays a pivotal role in the development of the prostate. The retinoic acid (RA) pathway has been reported to increase the budding of the prostate at the embryonic stage in a concentration-dependent manner, while simultaneously inhibiting the growth and branching of postnatal prostate ducts [15]. In conjunction with androgens, RA specifies the location and initiation of bud development in UGS epithelial cells and can inhibit the signaling transduction of Activin A produced by UGM [16]. Additionally, keratinocyte growth factor (KGF) is expressed in the developing prostate mesenchyme and probably promotes the morphological development of prostatic ducts by facilitating epithelial proliferation based on primary culture of isolated prostatic cells [17].

Our current understanding on the prostate is primarily based on rodent animal models and certain prostate cancer cell lines. The signal pathways during prostate development between humans and rodents are somehow conserved and both organs serve similar functions in reproduction, however, they exhibit significant differences in anatomical and histological aspects [18]. The human prostate is nearly mature at birth, contrasting with rodents that undergo secondary development during puberty. Additionally, while human prostatic cancer cell lines facilitate pathological research and drug screening, they cannot replicate the entire process of carcinogenesis. Moreover, human prostatic tissues are not easily accessed and there are various ethical constraints, leading to an extreme shortage of prostate research models. Therefore, establishing a human-derived prostate model is emergingly required for both basic and translational and clinical studies [19]. Human pluripotent stem cells (PSCs), including embryonic stem cells (ESCs) and induced pluripotent stem cells (iPSCs), have the potential to differentiate into all embryonic tissues and organs, which could closely mimic the signaling regulation and gene expression dynamics occurring during development [20]. Indeed, several studies have attempted this direction. Inspired by the pioneer study forty years ago that the tissue recombination of human urinary bladder epithelium with adult rat and mouse UGM led to the formation of prostatic acini after 30 days of cultivation [21], in 2006 scientists attempted to mix human ESCs with mouse UGM and then encapsulate them under the renal capsule of mice, resulting in the formation of prostatic tissue several months later [22]. Later in 2020, a study reported that human iPSC-derived definitive endoderm (DE) cells could form prostate organoids after 5 weeks’ co-culture with mouse UGM [23]. Similarly, a recent study revealed that DE cells, when co-cultured with spermatic vesical mesenchymal (SVM) cells derived from neonatal mice, could form prostate organoids [24]. These studies have demonstrated that human PSCs possess the potential to differentiate into prostatic lineage. However, previous reports rely on in vivo environment or the supporting mesenchymal cells, such as UGM or SVM, and the experimental process is complex and time-consuming, making it challenging to study the detailed molecular mechanism or use as disease models.

In this study, we are aiming to develop a differentiation protocol to generate human prostate organoids without supporting cells. We first follow the key developmental events during prostatic organogenesis to design a stepwise protocol to differentiate human ESCs into prostatic progenitors. We also apply the genetic manipulation technique to force NKX3-1 expression at specific stage. Finally, we explore the 3D culture to obtain prostate organoids in vitro.

## Materials and methods

### Cell cultures and differentiation

Human ESC lines HUES8 (obtained from Harvard Stem Cell Institute) and H9 (from Wicell Research Institute) [25, 26] were plated and expanded with mTeSR1 medium (STEMCELL Technologies, #85850) in Matrigel-coated (BD Bioscience, #354277) 6-well plates at the density of 100k cells per well. Culture medium was refreshed daily, and the cells were passaged every 4–5 days by accutase (Sigma, #A6964).

For differentiation, human ESCs were seeded in growth factor reduced Matrigel-coated (BD Bioscience, #354230) 24-well plates at a density of 60k cells/well with mTeSR1 medium added. For definitive endoderm differentiation, RPMI 1640 medium (Gibco, #C11875500BT) supplemented with 0.2% Bovine serum albumin (Yeasen, #36101ES76), 1% B27 supplement (without vitamin A) (Shanghai BasalMedia Technologies, #S441J7), 1% Penicillin-Streptomycin (Gibco, #15140163) was used. 100 ng/mL Activin A (PeproTech, #120-14b-1000) and 3 µM CHIR (Selleck, #S2924) were added at day 1, followed by 100 ng/mL Activin A alone for the following 2 days. For following hindgut and urogenital sinus cells differentiation, we mixed IMDM (Gibco, #C12440500BT) and F12 basic medium (Gibco, #C11765500BT) in a 1:1 ratio, supplemented with 0.2% Bovine serum albumin, 1% B27 supplement, 1% Penicillin-Streptomycin as basal medium. 3 µM CHIR and 100 ng/mL bFGF (PeproTech, #100-18b-500) were added to the basal medium for 2 days in hindgut differentiation. 3 µM CHIR, 1 µM TTNPB (Selleck, #S4627), 100 ng/mL KGF (PeproTech, #100-19-1000) and 1 µM DHT (Selleck, #S4757) were added to the basal medium for 3 days in UGS differentiation. The composition of the 3D culture medium is the same as the medium for differentiation from hindgut to urogenital sinus cells or based on prostate organoids culture medium used in previous studies [27, 28], supplemented with 2 µg/mL Doxycycline (Doxy) (Sigma-Aldrich, #324385).

For making prostate organoids, UGS cells were digested and collected using TrypLE solution (Gibco, #12604021), and resuspended with the cell density 800/µL. 25 µL of cell dilution and 50 µL of growth factor reduced Matrigel were mixed on ice, then the mixed solution was pipetted and dropped into a 24 well-plate, inverted in an incubator at 37 ℃ for 30 min to solidify, and then the 3D culture medium was added.

### Establishment of T-iNKX3-1 and AAVS1-iNKX3-1 cell lines

We obtained *NKX3-1* CDS sequence from cDNA, using the specific primers (5’-3’): ATGCTCAGGGTTCCGGAGCC, CCAAAAAGCTGGGCTCCAGC.

For transgenic T-iNKX3-1 cells, the pCW-Tet-puro-mCherry was cut with NheI restriction endonuclease, and the *NKX3-1* CDS was ligated, constructing the pCW-Tet-NKX3-1-puro-mCherry plasmid. Then, this vector was co-transfected with lentivirus packaging plasmids into HEK293T cells to make viral particles. The HUES8 cells infected with lentivirus were selected by puromycin. After 2 days of Doxy induction, NKX3-1 protein can be expressed together with the red fluorescence tag protein mCherry.

For knocked-in AAVS1-iNKX3-1 cells, we used *NKX3-1* CDS to replace dCas9 sequence of the Gen1 (pAAVS1-NDi-CRISPRi, Addgene #73497). The recombined vector, Gen1-NKX3-1, together with another plasmid pX459 (pSpCas9(BB)-2 A-Puro, Addgene #48139) containing sgRNA targeting AAVS1 locus [29], were electroporated into HUES8 cells using Nucleofector (Lonza). After 14 days of selection with 100 ng/mL G418, positive cells were purified. 2 µg/mL Doxy was used to induce the expression of NKX3-1 protein.

### Flow cytometry

Differentiated cells were digested into single cells by TrypLE and washed with DPBS containing 2% FBS. At the endoderm stage, single cell suspensions were stained using CXCR4-APC (1:200, BD Pharmingen, #555976) and APC-isotype (1:200, BioLegend, #400220) for 30 min. At the hindgut stage, the cells were incubated in Transcription Factor Buffer Set (BD Pharmingen, #562574) for 1 h before using CDX2 primary antibody (1:200, ZSGB-BIO, #ZA-0520) at 4 ℃ overnight and CY5-DoαRb secondary antibody (1:200, Jackson Immuno Research, #711-175-152) for 2 h in the dark at 4 ℃. Corresponding isotype was used as control. The method for cells isolated from prostate organoids is the same as above by using CK5 primary antibody (1:200, Servicebio, #GB111246-50), CK18 primary antibody (1:200, Servicebio, #GB12232-50), CY5-DoαMs secondary antibody (1:200, Jackson Immuno Research, #711-175-151) and FITC-DoαRb secondary antibody (1:200, Jackson Immuno Research, #711-095-152). The cells were detected by FACSCelesta flow cytometer (BD) or CytoFLEX flow cytometer (Beckman Coulter) and analyzed by FlowJo software.

### RT-qPCR

Total RNA was reversely transcribed into cDNA using ABScript III RT Master Mix (ABclonal, #RK20428) for qPCR. qPCR reactions were performed with the SYBR Green qPCR Master Mix (Biomake, #B21203) on the Real-time quantitative PCR instrument. The primers used in RT-qPCR are listed as follows (5’-3’): *CXCR4*: TACACCGAGGAAATGGGCTCA, AGATGATGGAGTAGATGGTGGG; *FOXA2*: GGAGCAGCTACTATGCAGAGC, CGTGTTCATGCCGTTCATCC; *SOX17*: GCATGACTCCGGTGTGAATCT, TCACACGTCAGGATAGTTGCAGT; *NANOG*: CCCCAGCCTTTACTCTTCCTA, CCAGGTTGAATTGTTCCAGGTC; *OCT4*: CAAAGCAGAAACCCTCGTGC, TCTCACTCGGTTCTCGATACTG; *CDX2*: GAACCTGTGCGAGTGGATG, CGGATGGTGATGTAGCGAC; *FOXA1*: GCAATACTCGCCTTACGGCT, TACACACCTTGGTAGTACGCC; *HOXA13*: CACGAACCCTTGGGTCTTC, TCTTTGGGGCAGTACATTTGG; *HOXA11*: TCCCATTGAATCTCCTTTGC, ATTTTCCTTGTGCCCAGTTG; *AR*: GGTGAGCAGAGTGCCCTATC, GCAGTCTCCAAACGCATGTC; *NKX3-1*: GGGTCTTATCTGTTGGACTCTG, GACAGGTACTTCTGATGGCTGA; *SOX9*: AAGTCGGTGAAGAACGGGC, CCTGGGATTGCCCCGAGTG; *TP63*: AGCCAGAAGAAAGGACAGCAG, GGTTCGTGTACTGTGGCTCACTAA; *KRT5*: TTGGACCAGTCAACATCTCTGT, TAGCTTCCACTGCTACCTCCG; *GAPDH*: AATGAAGGGGTCATTGATGG, AAGGTGAAGGTCGGAGTCAA. *GAPDH* was used as the internal reference, and the data was calculated by 2–ΔΔct method.

### Immunostaining, imaging and quantification

Cells grown on Matrigel-coated plate and the frozen sections of organoids tissue were fixed using 4% PFA for 30 min at room temperature followed by blocking and permeabilizing with 10% donkey serum, 0.3% Triton-X 100 in PBS (antibody buffer). Primary antibodies were resuspended in antibody buffer and incubated at 4℃ overnight. After wash, secondary antibodies were added in the dark for 2 h. Finally, the cells were incubated with DAPI for 10 min and then imaged on fluorescence microscope (Olympus). The following primary antibodies were used in this study: OCT4 (1:200, CST, #2750S), SSEA4 (1:200, Millipore, #MAB4304), SOX17 (1:200, R&D, #AF1924), FOXA2 (1:200, CST, #3143S), CDX2 (1:200, ZSGB-BIO, #ZA-0520), FOXA1 (1:200, CST, #53528S), NKX3-1 (1:200, CST, #83700S), AR (1:200, CST, #5153S), P63 (1:200, CST, #13109), CK5 (1:200, CST, #25807), CK8/18 (1:200, CST, #4546), CK18 (1:400, Servicebio, #GB12232-50), E-Cadherin (CST, #3195S). The following secondary antibodies were used here: 488-DoαRb (1:200, Jackson Immuno Research, #711-545-152), 488-DoαMs (1:200, Jackson Immuno Research, #715-545-150), FITC-DoαRb (1:200, Jackson Immuno Research, #711-095-152), TRITC-DoαMs (1:200, Jackson Immuno Research, #715-025-150), CY5-DoαMs (1:200, Jackson Immuno Research, #711-175-151).

For confocal imaging, cells grown on Matrigel-coated plate were fixed with 4% PFA. The dome structure of organoids was scraped off with a spoon or scraper and the organoids were placed in a 1.5mL Eppendorf tube. Followed by washing with PBS and blocking with antibody buffer for 1 h at room temperature under constant rolling, we incubated the samples with primary antibodies overnight at 4 °C and secondary antibodies for 2 h in the dark at room temperature. Following PBS wash, samples were incubated with DAPI. The whole process of incubation was under constant rolling. We used the coated tip to resuspend organoids in 200 µL PBS and transferred them to 20 mm Glass Bottom Culture Dish. Confocal images were captured with the Leica STELLARIS 5 WLL Confocal Platform.

Using the Image J software, we obtained the number of target protein-positive cells and DAPI-positive cells respectively in each image. The positive percentage was further calculated by the ratio of target protein-positive cells over DAPI-positive cells. Finally, the average of positive percentages from 3 different images was used in the figure.

### H&E staining

We fixed organoid samples with 4% PFA for 1 h, wrapped group organoids with liquid agarose, allowed them to solidify and then fixed in 4% PFA overnight, and then dehydrated with a density gradient of ethanol and xylene. The tissues were then embedded in paraffin and sectioned, followed by dewaxing using xylene and ethanol. Finally, the nucleus and cytoplasm were stained with hematoxylin-eosin, the specimens were dehydrated, sealed, and observed under a microscope (Leica Aperio VERSA 8).

### Western blot

Cells were lysed with RIPA buffer containing phosphatase inhibitor and protease inhibitor for 45 min on ice and then centrifuged at 12,000 g at 4 °C for 10 min. The supernatant was then removed, and 5× loading buffer was added and denatured at 100 °C for 10 min. Protein lysates were loaded on SDS-PAGE gels and electro-transferred onto polyvinylidene fluoride (PVDF) membrane. The samples were blocked by 5% fat-free milk solution TBST for 2 h at room temperature and exposed to primary antibodies overnight at 4 °C. HRP-conjugated secondary antibodies were used for 2 h at room temperature. The protein bands were visualized using an enhanced chemiluminescence (ECL) kit and the BioSpectrum^®^ 515 Imaging System was used for data processing. The following primary antibodies were used in this study: AR (1:1000, Santa Cruz, #N-20), FOXA1 (1:1000, CST, #53528S), NKX3-1 (1:1000, CST, #83700S), β-Actin (1:1000, Santa Cruz, #sc-47778). The following secondary antibodies were used: Anti-Mouse-IgG (H + L)-HRP (1:1000, Sungene Biotech, #LK2003), Anti-Rabbit-IgG (H + L)-HRP (1:1000, Sungene Biotech, #LK2001).

### Statistical analyses

Data are presented as mean ± SEM. The unpaired t test was applied for statistical analyses of differences between two groups, and differences with values of *p* < 0.05 were considered statistically significant. ns, not significant (*p* > 0.05), *p < 0.05, **p < 0.01, ***p < 0.001.

## Results

### Highly efficient differentiation of definitive endoderm and hindgut cells from ESCs

**Fig. 1:**
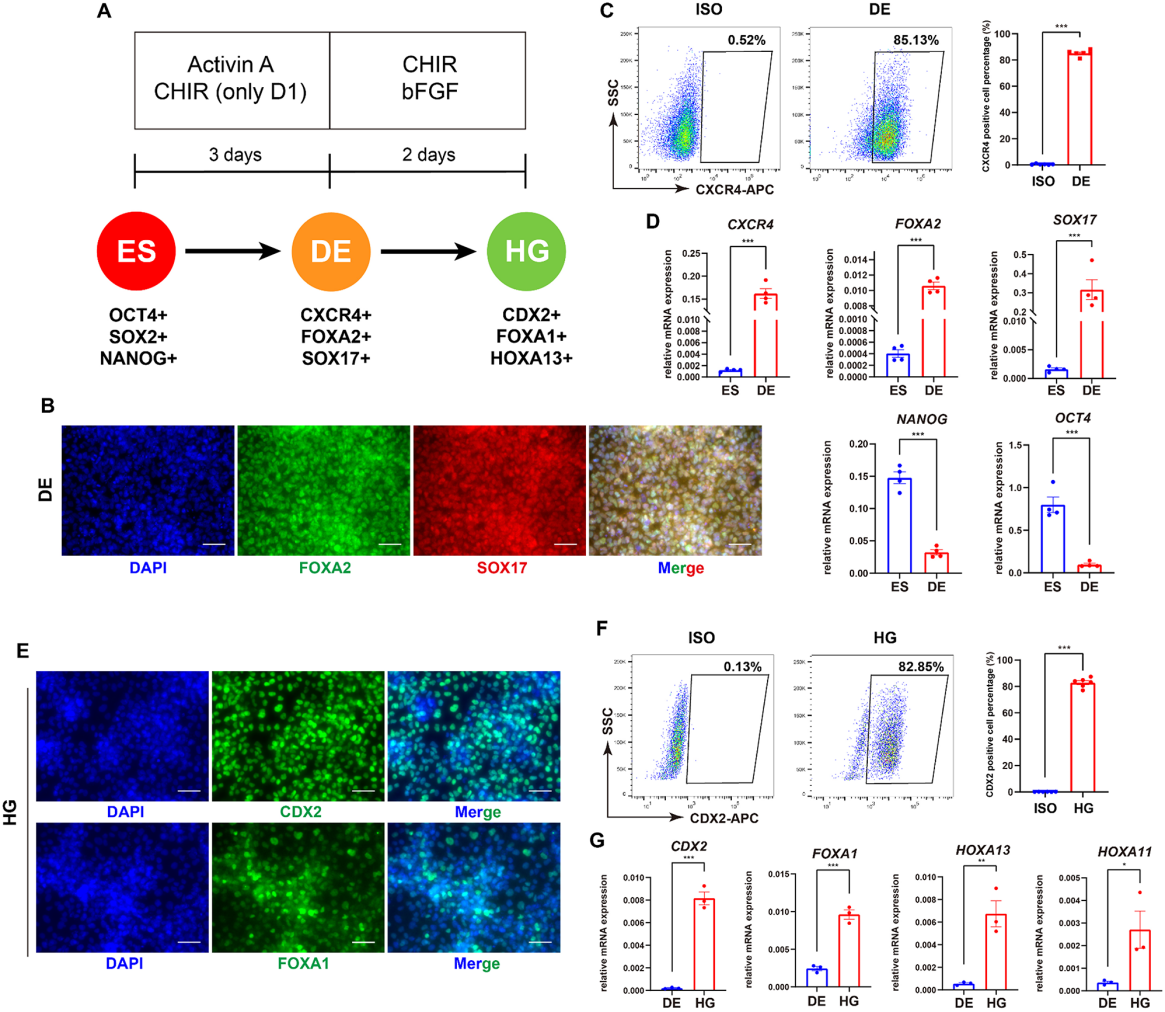
Generation of definitive endoderm and hindgut cells from human ESCs. (**A**) Schematic diagram of the differentiation of human ESCs towards the hindgut lineage, along with the small molecules required for this process, and markers at each stage of the differentiation (ES: human embryonic stem cell, DE: definitive endoderm cell, HG: hindgut cell, ISO: isotype control). (**B**) Immunofluorescence results for endodermal factors SOX17 and FOXA2 at DE stage. Scale bar = 50 μm. (**C**) Flow cytometry results of CXCR4 at DE stage, along with the quantitative analysis (*n* = 6). (**D**) Changes in mRNA relative expression levels during the DE differentiation (*n* = 4). (**E**) Immunofluorescence results for CDX2 and FOXA1 at hindgut stage. Scale bar = 50 μm. (**F**) Flow cytometry results for CDX2 expression at hindgut stage, along with the quantitative analysis (*n* = 6). (**G**) Changes in mRNA relative expression levels during hindgut differentiation (*n* = 3). *p < 0.05, **p < 0.01, ***p < 0.001

The prostate gland arises from the urogenital sinus (UGS), which is derived from the tail extension of the hindgut derived from the endoderm [3, 30]. Therefore, accordingly divided our protocol into different stages: definitive endoderm (DE), hindgut (HG) (Fig. 1A), and then UGS and prostatic lineages. First, based on our recent studies about DE differentiation [31, 32] and other literatures [33, 34], we used the human ESC line HUES8 to generate DE, which was achieved by adding CHIR and Activin A to activate the WNT signaling pathway and TGFβ signaling pathway for 1 day, and solely adding Activin A to activate the TGFβ signaling pathway in next 2 days. The DE differentiation was then evaluated by gene expression analysis. At the protein level, immunofluorescence results indicated that key transcription factors FOXA2 and SOX17 were positively stained in the generated DE cells (Fig. 1B), and flow cytometry detected an 85.13% ± 0.95% positive rate for the surface marker CXCR4 in these cells (Fig. 1C). Meanwhile, the RT-qPCR assay revealed significant up-regulation of endoderm genes (*SOX17*, *FOXA2*, *CXCR4*) and significant down-regulation of pluripotency genes (*NANOG* and *OCT4*) after differentiation (Fig. 1D). These data indicate that our protocol can yield definitive endodermal cells with high efficiency.

The process of posterior hindgut differentiation from DE is primarily regulated by WNT and FGF signaling pathways [35]. Therefore, we applied CHIR and bFGF to activate the WNT and FGF signaling pathways, respectively, to generate hindgut cells. After 2 days’ treatment, the immunofluorescence results indicated that the two key transcription factors determining the fate of posterior gut cells, CDX2 and FOXA1, were positive (Fig. 1E). Flow cytometry results showed that the percentage of CDX2-positive cells reached 82.85% ± 1.49% (Fig. 1F). RT-qPCR results further showed that the expression levels of hindgut marker genes *CDX2*, *FOXA1*, *HOXA13* and *HOXA11* increased significantly compared to DE cells (Fig. 1G). These data collectively show that the hindgut cells are efficiently generated.

Furthermore, to validate the effectiveness of our protocol, we applied the same differentiation protocol in another ESC line, H9 (WA-09). Flow cytometry analysis revealed that the positive rate of cells at the definitive endoderm and hindgut stages both exceeded 90% (Figure S1A-B), confirming that this protocol can successfully induce ESCs to differentiate into definitive endoderm and hindgut cells.

### Generation of AR and FOXA1 double-positive UGS cells

**Fig. 2:**
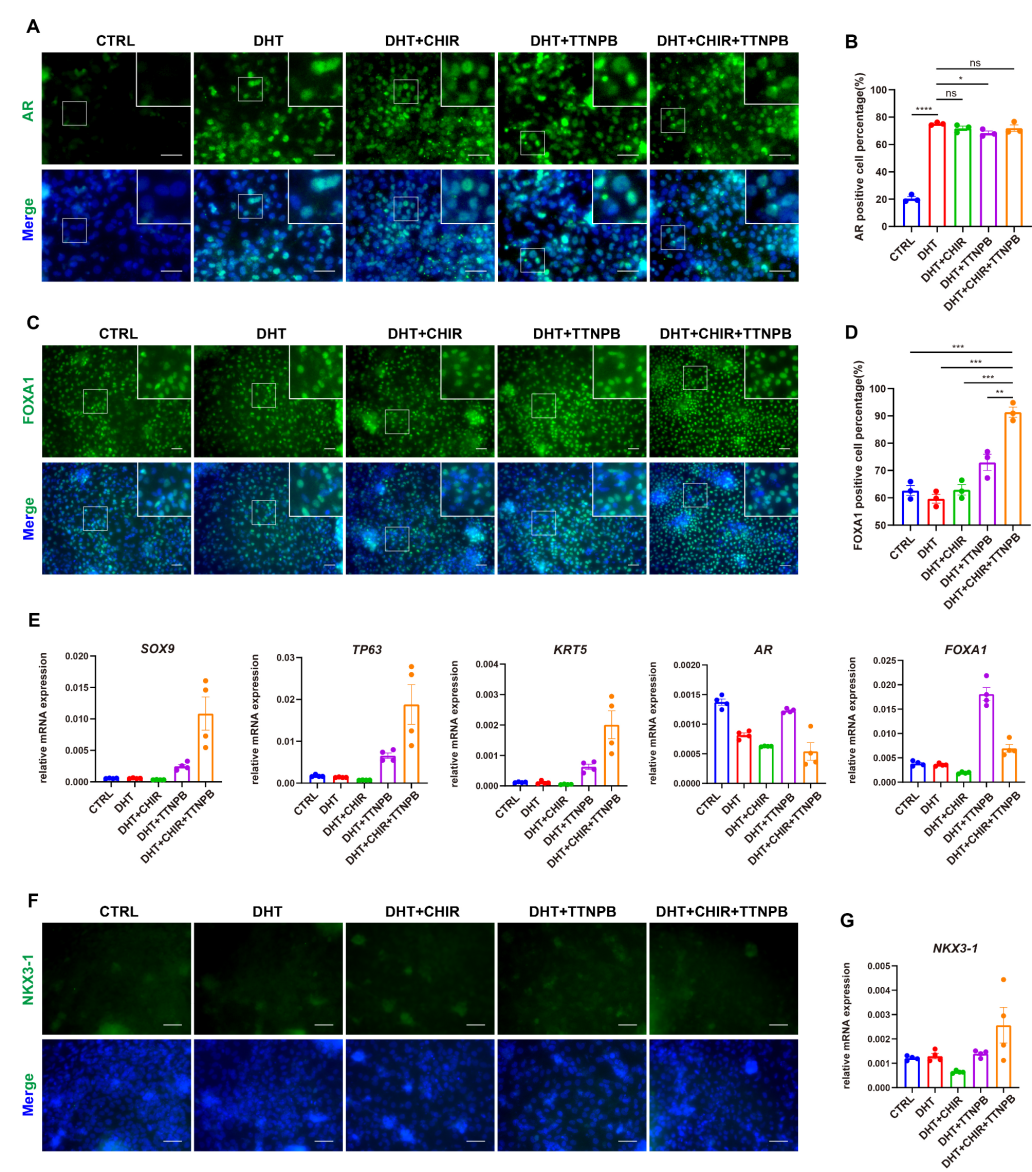
Generation of AR and FOXA1 double-positive UGS cells. (**A**) The immunofluorescence results of AR after differentiation with different conditions. Scale bar = 50 μm (CTRL: control). (**B**) Statistical results of AR-positive cells in (**A**) (*n* = 3). (**C**) The immunofluorescence results of FOXA1 after differentiation with different conditions. Scale bar = 50 μm. (**D**) Statistical results of FOXA1-positive cells in (**C**) (*n* = 3). (**E**) Changes in the mRNA levels of the prostatic markers *SOX9*, *P63* (*TP63*), and *CK5* (*KRT5*) after differentiation with different conditions (*n* = 4). (**F**-**G**) The immunofluorescence results (**F**) and mRNA levels (**G**, *n* = 4) of NKX3-1 after differentiation. Scale bar = 50 μm. ns, not significant (*p* > 0.05), *p < 0.05, **p < 0.01, ***p < 0.001

For UGS induction from hindgut cells, we utilized DHT, TTNPB, and CHIR to activate the AR, RA, and WNT signaling pathways, respectively. Meanwhile, we also introduced the growth factor KGF to promote cell survival and growth. We titrated the concentrations of individual factor and compared the induction efficacy of different combinations. The immunofluorescence results indicated that the AR-positive cells were significantly increased upon DHT treatment, while other conditions had no significant effect (Fig. 2A-B). For FOXA1, immunofluorescence results suggested that the combination of AR, RA and WNT pathways maximized the percentage of FOXA1-positive cells (Fig. 2C-D). RT-qPCR assay confirmed that the combination led to increased levels of *SOX9*, *TP63*, and *KRT5* genes, markers of UGS and prostatic lineage (Fig. 2E), albeit *AR* and *FOXA1* did not show significant increases at the mRNA level. However, this protocol failed to yield significant NKX3-1-positive cells, shown by the immunofluorescence result (Fig. 2F), although RNA analysis showed only a little increase of *NKX3-1* expression upon the combined treatment (Fig. 2G). The results from another ESC line H9 further confirmed that our protocol could effectively induce the generation of AR and FOXA1 double positive cells but failed to stimulate the appearance of NKX3-1-positive cells (Figure S1C).

Since the NKX3-1-positive cells was not induced through the chemicals and NKX3-1 is essential to promote prostatic lineage differentiation, we considered to force the expression of NKX3-1 by genetic manipulation. Therefore, we constructed a lentiviral plasmid with a Tet cassette (i.e., pCW-Tet-NKX3-1-puro-mCherry) and then the packaged viral particles were infected to HUES8 cells. After selection with puromycin, we obtained a transgenic NKX3-1-inducible ESC line (named as T-iNKX3-1) (Figure S2A). This line maintained typical ESC-like colonies and normally expressed the pluripotency markers OCT4 and SOX2, checked by immunostaining (Figure S2B). Then, we subjected this cell line to prostatic differentiation using our protocol. On day 3 of differentiation, flow cytometry results showed more than 90% CXCR4-positive DE cells (Figure S2C); On day 5, there were more than 85% CDX2-positive cells (Figure S2D), indicating the efficient differentiation of DE and hindgut cells. Subsequently, Doxy was added along with the differentiation medium to induce NKX3-1 overexpression during UGS induction. However, the immunofluorescence results suggested that Doxy treatment only promoted a small number, less than 5%, of cells expressing NKX3-1 (Figure S2E).

To understand the low efficiency of overexpression, we went back to check the cell line T-iNKX3-1. Brightfield and immunofluorescence images showed that not all the cells could express mCherry (Figure S2F). Only around 20% cells exhibited obvious mCherry signal (Figure S2G). In consistent, only a small subset of cells expressed NKX3-1 after Doxy treatment (Figure S2H). This indicates that the inducible expression is achievable in human ESCs, but the transgenic approach fails to stimulate high levels of NKX3-1 overexpression during differentiation.

### Establishment and characterization of the knock-in NKX3-1-inducible ESCs

**Fig. 3:**
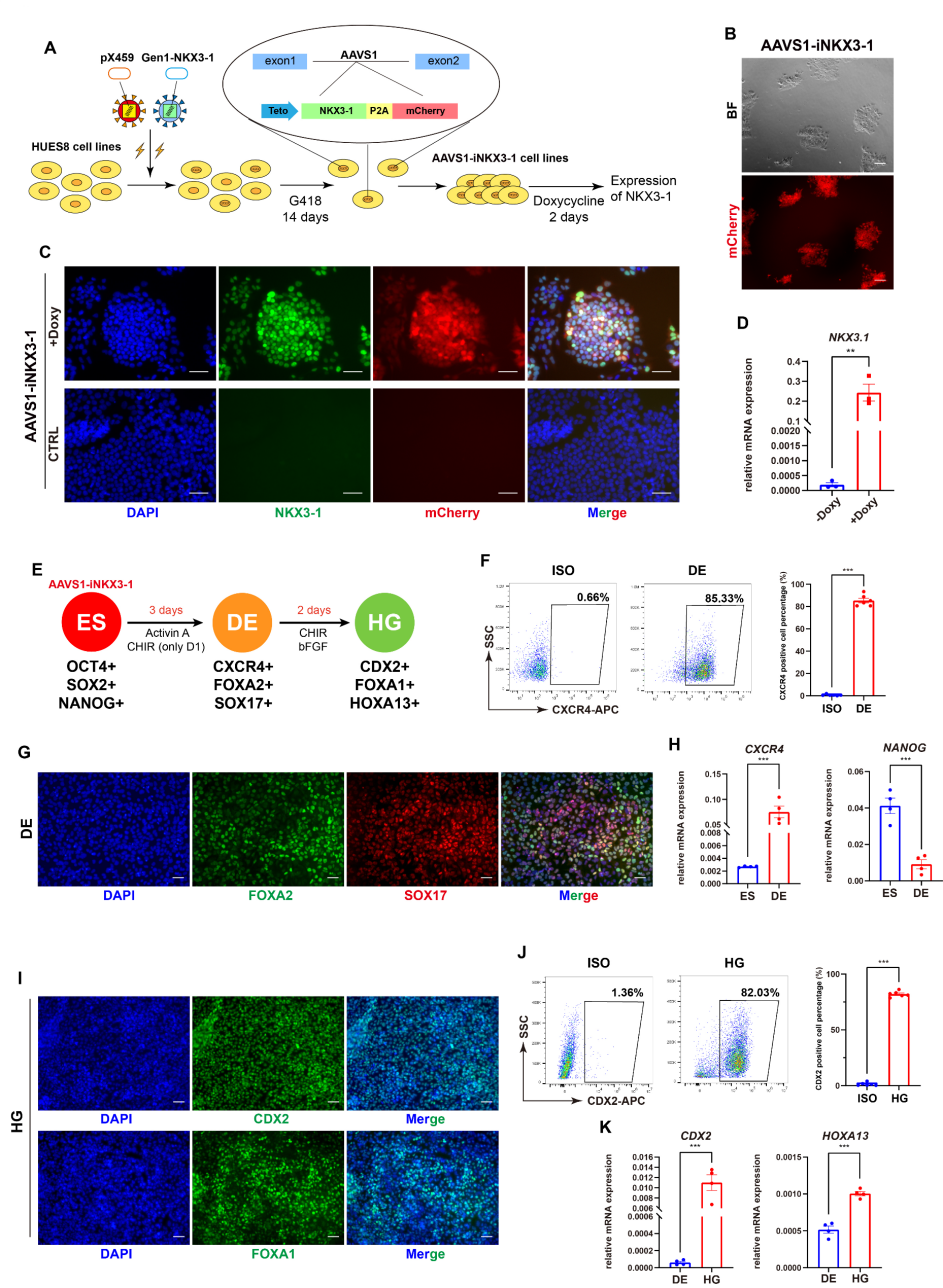
Construction and hindgut differentiation of AAVS1-iNKX3-1 ESC line. (**A**) Schematic diagram of knocking NKX3-1 expressing cassette into AAVS1 site. (**B**) Brightfield and mCherry immunofluorescence results of AAVS1-iNKX3-1 cells. Scale bar = 50 μm (BF: Brightfield). (**C**) Immunofluorescence image of NKX3-1 and mCherry after Doxy induction for 48 h in AAVS1-iNKX3-1 cells, with the control without Doxy induction (CTRL: control, Doxy: Doxycycline). (**D**) Relative mRNA expression of *NKX3-1* after Doxy induction for 48 h in AAVS1-iNKX3-1 cells (*n* = 3). (**E**) Schematic diagram of hindgut differentiation process (ES: human embryonic stem cell, DE: definitive endoderm cell, HG: hindgut cell). (**F**) Flow cytometry results of CXCR4 at the DE stage, as well as the quantification (*n* = 6) (ISO: isotype control). (**G**) Immunofluorescence image of SOX17 and FOXA2 at the DE stage. (**H**) Changes in relative mRNA expression during DE differentiation (*n* = 4). (**I**) Immunofluorescence image of CDX2 and FOXA1 at the HG stage. (**J**) Flow cytometry results of CDX2 at the HG stage, as well as the quantification (*n* = 6). (**K**) Changes in relative mRNA expression during hindgut differentiation (*n* = 4). **p < 0.01, ***p < 0.001

The failure of T-iNKX3-1 promoted us to consider knocking the NKX3-1 expression caste into the safe harbor site to prevent the transgenic silence in ESCs. Therefore, based on our recent experience [29], we first made Gen1-NKX3-1 vector and pX459 containing Cas9 and guide RNA targeting endogenous AAVS1 site. After transfection followed by 14 days’ selection with G418, we obtained the knocked-in inducible NKX3-1 ESC line (named as AAVS1-iNKX3-1) (Fig. 3A). Brightfield and immunofluorescence images showed that this cell line remained typical undifferentiated pluripotent colonies and expressed mCherry upon Doxy induction (Fig. 3B). Flow cytometry analysis indicated that over 95% of the cells expressed mCherry (Figure S3A), significantly higher than the previous T-iNKX3-1 cell line (Figure S2G). Immunofluorescence results suggested that the pluripotency markers OCT4 and SSEA4 were still highly expressed (Figure S3B), consistent with the colony morphology. The AAVS1-iNKX3-1 cells indeed expressed NKX3-1 upon Doxy treatment and without Doxy induction they did not express NKX3-1 or mCherry (Fig. 3C). RT-qPCR results also confirmed the overexpression of *NKX3-1* upon Doxy induction (Fig. 3D). These data confirmed the induction of NKX3-1 by Doxy, thereby serving as the basis for subsequent experiments for differentiation.

We then subjected the AAVS1-iNKX3-1 cell line to prostatic differentiation with the same differentiation protocol (Fig. 3E). We detected more than 85% of CXCR4-positive cells at DE stage (Fig. 3F), which was further validated by immunofluorescence of FOXA2 and SOX17 (Fig. 3G) and RNA analysis that upregulated *CXCR4* gene and downregulated *NANOG* (Fig. 3H). At hindgut stage, we performed immunofluorescence assay and the results showed that both the hindgut markers CDX2 and FOXA1 were positive (Fig. 3I). Flow cytometry indicated that the percentage of CDX2-positive cells was over 80% (Fig. 3J), and RT-qPCR results suggested that the expression levels of the hindgut markers *CDX2* and *HOXA13* were significantly elevated (Fig. 3K). These data further confirmed that AAVS1-iNKX3-1 cell lines maintained the normal hindgut differentiation ability.

### Induction of UGS and prostatic cells from the AAVS1-iNKX3-1 cells

**Fig. 4:**
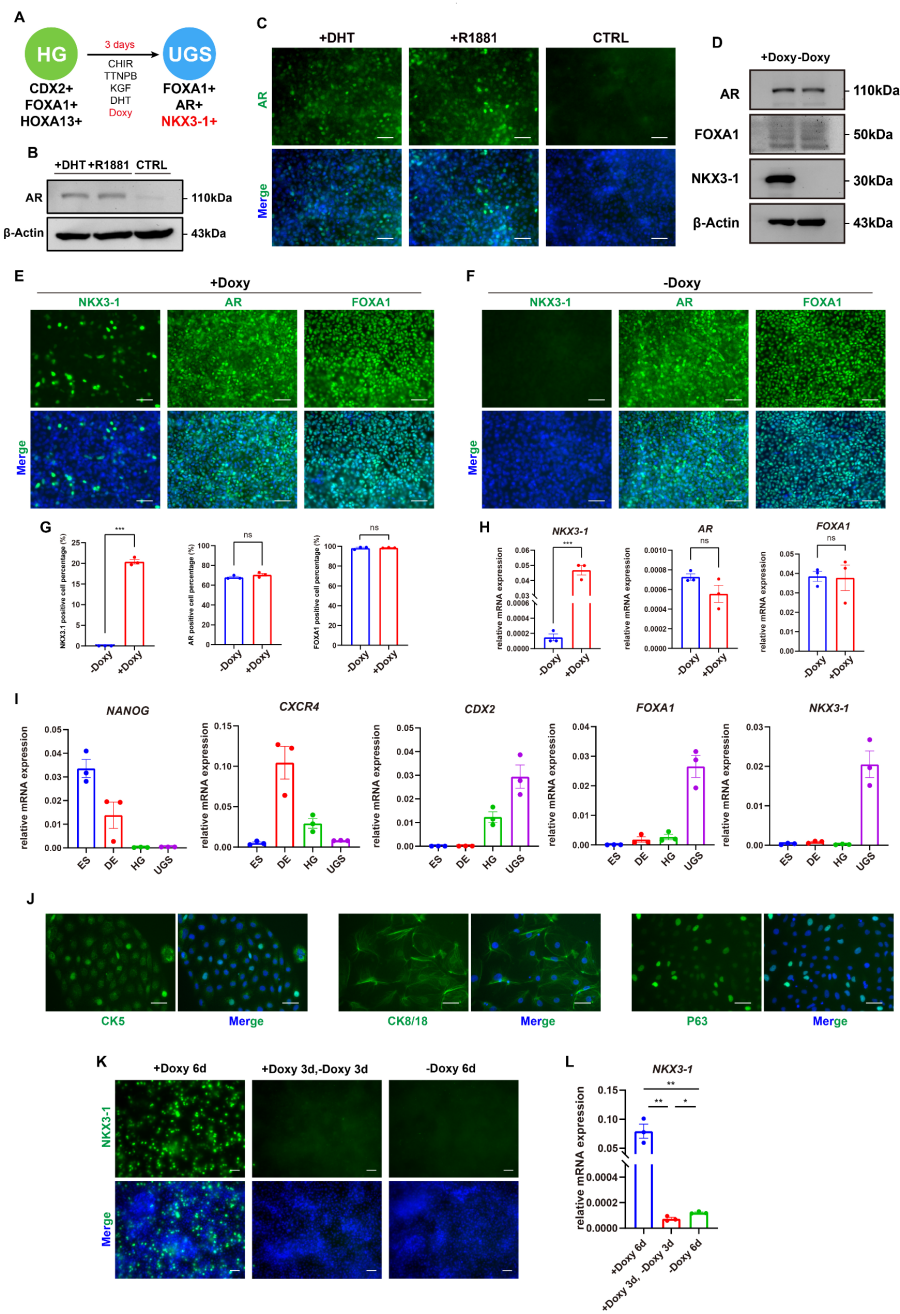
Differentiation of NKX3-1, AR, and FOXA1 triple-positive UGS cells. (**A**) Schematic diagram of the UGS differentiation from hindgut (HG: hindgut cell, UGS: Urogenital sinus cell, Doxy: Doxycycline). (**B**-**C**) Western blot results (**B**) (Full-length blots/gels are presented in Supplementary Fig. 4A) and immunofluorescence images (**C**) of AR protein in cells treated with DHT, R1881, and without androgen induction as control during UGS differentiation. The molecular weight of the protein was labeled at right. Scale bar, 50 μm (CTRL: control). (**D**-**F**) Western blot results (**D**) (Full-length blots/gels are presented in Supplementary Fig. 4B) and immunofluorescence images (**E**-**F**) of AR, FOXA1 and NKX3-1 proteins in cells treated with (**E**) or without (**F**) Doxy during UGS differentiation. The molecular weight of the protein was labeled at right. Scale bar, 50 μm. (**G**) Statistical analysis of the percentages of positive cells for NKX3-1, AR, and FOXA1 with or without Doxy induction during UGS differentiation (*n* = 3). (**H**) Relative mRNA expression levels of *NKX3-1*, *AR*, and *FOXA1* with or without Doxy induction during UGS differentiation (*n* = 3). (**I**) Changes in relative mRNA expression levels of markers at various stages throughout the differentiation process from ESCs to UGS (*n* = 3) (ES: human embryonic stem cell, DE: definitive endoderm cell). (**J**) Immunofluorescence results of CK5, CK8/18, and P63 in UGS cells. Scale bar, 50 μm. (**K**-**L**) Immunofluorescence images (**K**) and relative mRNA expression level (**L**) of *NKX3-1* after adding Doxy induction for 6 days, for 3 days’ treatment followed by 3 days’ withdrawal, and without Doxy induction for 6 days during UGS differentiation (*n* = 3). Scale bar, 50 μm. ns, not significant (*p* > 0.05), *p < 0.05, **p < 0.01, ***p < 0.001

Next, we wondered whether the hindgut derived from the AAVS1-iNKX3-1 cells could further differentiate into UGS and prostatic lineage cells. We treated the hindgut with Doxy to force the expression of NKX3-1 based on previous condition (Fig. 4A). Initially, we compared the response of two forms of androgens, either natural androgen DHT or synthetic androgen R1881, in this cell line. Western blot result indicated that both DHT and R1881 enhanced AR protein expression, whereas AR protein expression was virtually absent in the absence of androgens (Fig. 4B). Immunofluorescence results demonstrated that both DHT and R1881 promoted numerous AR-positive cells, with nuclear localization, consistent with the notion that androgens facilitate AR nuclear entry (Fig. 4C). Next, we evaluated the effect of Doxy as well as the UGS differentiation. Western blot analysis revealed that the addition of Doxy greatly increased the expression of NKX3-1, albeit no obvious impact on AR and FOXA1 protein expression (Fig. 4D). Immunofluorescence results showed that cells induced with Doxy could express AR, FOXA1 and NKX3-1 (Fig. 4E), representing the UGS and prostatic lineage. In contrast, cells differentiation without Doxy induction could only yield AR and FOXA1 double-positive cells (Fig. 4F). By analyzing the positive rates of cells in the immunofluorescence results, the NKX3-1-positive cells reached 20%, and the AR-positive cells and FOXA1-positive cells were higher than 60% and 90%, respectively (Fig. 4G). RT-qPCR analysis revealed that the mRNA expression of *NKX3-1* increased significantly after Doxy induction, despite no difference in mRNA expression of *AR* and *FOXA1* (Fig. 4H).

Since the AR, FOXA1 and NKX3-1 triple-positive cells were successfully generated from human ESCs, we examined the RNA expression dynamics during the entire differentiation process from ESCs to UGS stage under the current protocol. It is evident that the pluripotent gene *NANOG* was continuously down-regulated, while the endoderm gene *CXCR4* was most highly expressed at DE stage and then down-regulated along with the further differentiation. The expression of hindgut gene *CDX2* exhibited a significant increase, starting from the hindgut stage and with further elevation during the subsequent UGS stage. Importantly, the expression levels of the prostate markers *NKX3-1* and *FOXA1* reached the peak at UGS stage (Fig. 4I).

In addition, CK5 and P63 as markers for the prostatic basal epithelium, and CK8/18 as marker for the luminal epithelium start to express during the pre-budding phase from the UGS [1]. Therefore, we examined these markers in our differentiated UGS cells. Consistently, we observed the presence of CK5 and CK8/18 localized to the cytoplasm, as well as P63-positive cells located in the nucleus (Fig. 4J). These data collectively suggest the appearance of prostatic intermediate cells positive for AR, FOXA1, NKX3-1, CK5, CK8/18, and P63.

We examined whether Doxy treatment could drive the endogenous expression of NKX3-1, thereby facilitating intrinsic prostatic differentiation. Therefore, starting from the differentiation towards the prostate, we tested three different treatments at UGS stage: continuous Doxy induction for 6 days, continuous induction for 3 days followed by withdrawal for another 3 days, and no Doxy induction for 6 days. The results revealed that only the continuous treatment of Doxy had obvious NKX3-1-positive cells, while none of NKX3-1-positive cells were observed in the other two groups (Fig. 4K). RT-qPCR analysis confirmed this result that continuous Doxy induction for 6 days exhibited the highest mRNA level of *NKX3-1* (Fig. 4L).

### Prostatic organoids formation through 3D cultivation

**Fig. 5:**
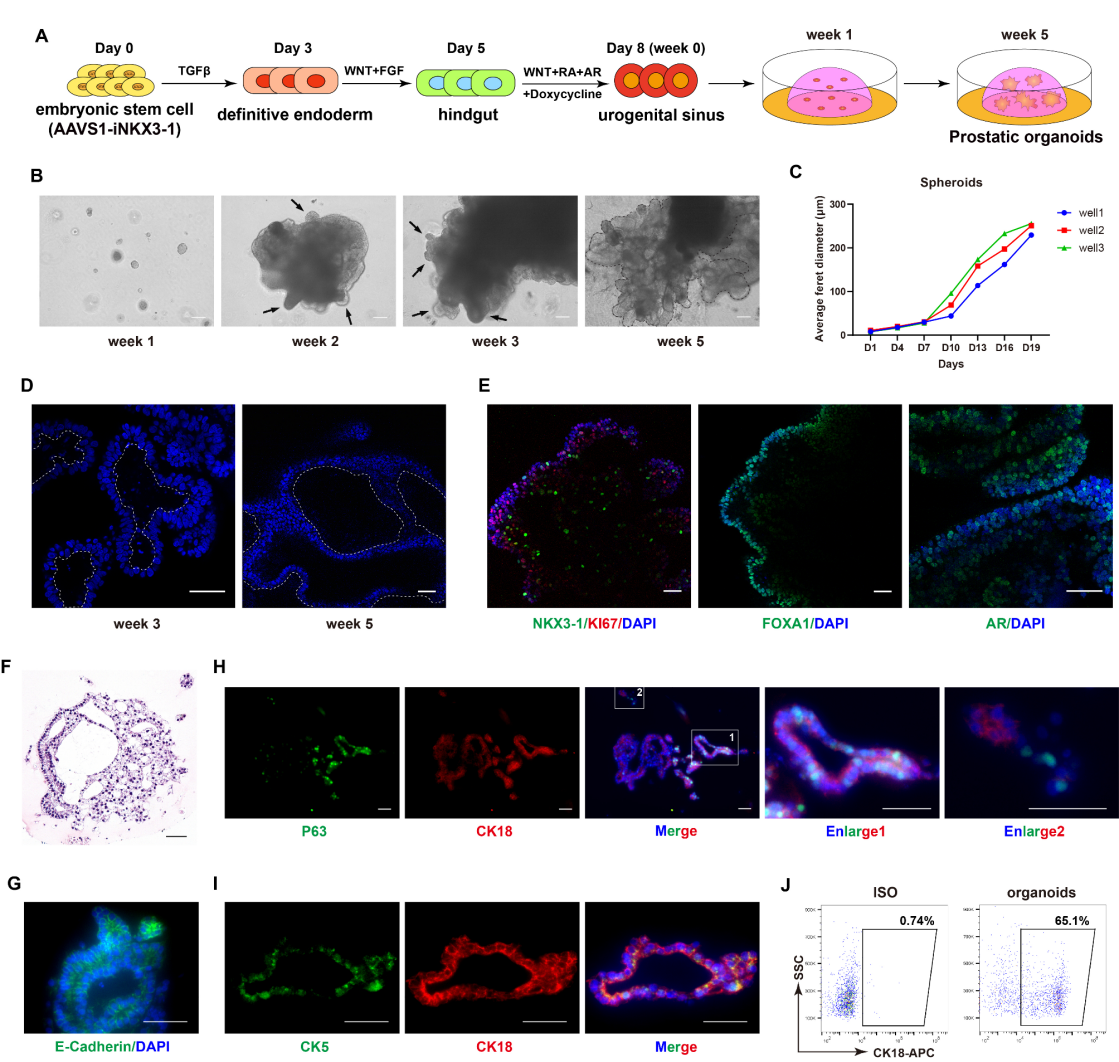
The formation of structural prostate organoids through 3D culture of ESC-derived UGS cells. (**A**) Illustration of the generation of prostatic organoids from human ESCs. (**B**) Brightfield images of organoids at week 1, 2, 3, and 5. Scale bar, 50 μm. (**C**) Diameter statistical graph of organoids after 3D cultivation for the first 20 days. (**D**) DAPI immunofluorescence results of organoids on week 3 and 5. Scale bar, 50 μm. (**E**) Immunofluorescence results of NKX3-1, KI67, FOXA1, and AR on week 3 of organoids cultivation. Scale bar, 50 μm. (**F**-**I**) H&E staining images (**F**), immunofluorescence results of E-Cadherin (**G**), P63 and CK18 (**H**), and CK5 and CK18 (**I**) of organoids at week 4. Scale bar, 50 μm. (**J**) Flow cytometry results of CK18 of organoids. 3D cultured organoids for 9 days were used (ISO: isotype control)

We induced differentiation of human embryonic stem cell lines by activating relevant pathways and obtained the urogenital sinus cells through the definitive endoderm and hindgut stages. Since the extracellular matrix (ECM) functions as a supportive structure for prostate, we thus utilized Matrigel to embed the generated UGS cells for further differentiation and organoids formation (Fig. 5A). The brightfield images showed that the organoids could form but retaining a spherical structure with a relatively small volume after one week of 3D culture; by week 2, the organoids were gathered into spherical-shaped protrusions at localized locations; by week 3, while the volume of the organoids was increased, several bud-like structures were also observed; by week 5, more complex curved tubular structures were observed at the sites of the buds (Fig. 5B). The serial of observation suggested that throughout the 3D cultivation process, the organoids might not only undergo significant size increase but also gradually specialize from a spherical form into more complex stripes and tubular lumens (Fig. 5B). With diameters below 50 μm on day 7, the organoids exhibited a significant increase in growth rate after day 7, exceeding 100 μm on day 13, and surpassing 200 μm on day 19. Statistical results on the average diameter of the organoids confirmed the observation that the organoids continued to grow (Fig. 5C). The nuclei staining on week 3 and week 5 showed that the organoids gradually emerged larger tubular structures (Fig. 5D). Meanwhile, the KI67 staining of organoids further indicated that the prostatic lineage cells were somehow proliferative and most cells within the organoids maintained positive for AR and FOXA1 (Fig. 5E).

To validate that the organoids indeed represent prostate lineage and structure, we first conducted H&E staining, which indicated the organoids on week 4 showed that they displayed a ductal structure resembling that of the normal prostates with luminal structures in organoids (Fig. 5F) [36, 37]. And the main components of the organoids were epithelial cells with the E-Cadherin staining (Fig. 5G). Subsequently, we found that these organoids contained a substantial population of luminal cells positive for CK18 (Fig. 5H), with a subset of intermediate cells co-expressing luminal epithelial marker CK18 and basal epithelial marker P63 (Fig. 5H) [38, 39]; in addition, the basal epithelial cells solely expressing P63 could also be visualized (Fig. 5H). We also co-stained with CK18 and CK5, again indicating that these organoids contained a substantial population of luminal cells positive for CK18, with a subset of intermediate cells co-expressing luminal CK18 and basal CK5 (Fig. 5I). The flow cytometric analysis that approximately 65% cells were CK18-positive (Fig. 5J) together with the results above indicated that the organoids generated prostate luminal epithelium and corresponding structures, and the organoids predominantly consisted of luminal epithelial cells, with a minority intermediate cells and basal epithelial cells.

## Discussion

The present study reported an experimental protocol to induce human ESCs to differentiate into prostatic lineage (Fig. 5A), initially by applying relevant signaling pathways found from developmental studies and later combining the inducible overexpression of key factor NKX3-1. We established a useful human ESC line carrying an inducible NKX3-1 expression cassette inserting into AAVS1 site. Based on this cell line and the protocol, we were able to generate the AR, FOXA1 and NKX3-1 triple-positive UGS and prostatic progenitors, and further obtained prostatic organoids which were similar to the prostate in terms of structure and marker expression patterns through 3D culture. This study thus provides a feasible and alternative platform to study human prostate.

Our protocol has mimicked some major events during prostate development. Activin A and CHIR were used to activate the TGFβ and WNT pathways respectively to differentiate human ESCs into mesendoderm and further definitive endoderm stage; then, the FGF and WNT pathways activated by bFGF and CHIR, respectively, to induce DE into HG cells (Fig. 1). Afterwards, we examined the effects of AR, RA, and WNT agonists on UGS and prostatic lineage differentiation and obtained AR and FOXA1 double-positive cells (Fig. 2), which are key factors for prostatic lineage [40]. To overcome the NKX3-1 issue, we constructed two different *NKX3-1* inducible cell lines. Although the transgenic line based on lentiviral system exhibited exogenous transgene silence and failed to generate highly efficient NKX3-1-positive UGS cells, we eventually obtained AR, FOXA1 and NKX3-1 triple positive progenitors based on the knock-in AAVS1-iNKX3-1 cell line and these cells also expressed prostatic lineage markers CK5, CK8/18 and P63 (Fig. 4). Lineage studies indicate that P63 can maintain the prostate lineage and generate prostatic epithelial cells, and the absence of P63 restricts the differentiation of the urogenital sinus towards the prostate direction [41]. Additionally, CK5 and CK18 are co-expressed during the early stages of urogenital sinus budding, prior to the formation of a luminal structure (39). Finally, we employed a Matrigel-based 3D culture system and observed the formation of prostatic organoids, with significant size increasement and initiation of budding as well as evolving complex structures resembling lumens and epithelia. H&E and immunofluorescence staining results confirm that the organoids possess a luminal structure and express luminal epithelial markers such as CK18, AR and NKX3-1. Additionally, a proportion of cells co-expressed both CK18 and basal epithelial markers, P63 and CK5, indicating that the organoids obtained through our differentiation protocol have possessed the characteristics of a native prostate in certain aspects (Fig. 5). Taken together, we have successfully generated AR, FOXA1 and NKX3-1 triple-positive UGS cells and constructed a prostate organoid model.

The NKX3-1 protein may function as a transcription factor, and its expression is confined exclusively to the prostate, suggesting that the NKX3-1 gene may play a pivotal role in driving the differentiation of the prostate [9]. The protein is expressed at the rostral and caudal ends of the urogenital sinus epithelium during the early development of the prostate, corresponding to regions of active morphogenesis [10]. NKX3-1 is expressed uniformly in the epithelial cells, and later its expression is largely restricted to the luminal cells [10, 42]. DNA binding property of NKX3-1 is essential for specifying prostatic differentiation during embryological development [43]. NKX3-1 regulates the proliferation and differentiation of the prostate epithelium in a dose-sensitive manner [44]. The absence of this protein leads to downregulation of genes essential for prostate differentiation, reduction in duct branching, decreased secretion of proteins, as well as epithelial hyperplasia and dysplasia of the prostate [10]. In addition, NKX3-1 can interact with other factors during prostate development, promoting the morphological development of intraluminal branching and luminal differentiation [14]. For instance, β-catenin interacts with NKX3-1 to promote prostate bud development [45]. Additionally, NKX3-1 and Topoisomerase I are co-localized to the enhancer region of *AR* gene to mediate the transcription program of *AR* [46]. In conclusion, NKX3-1 is a prostate-specific transcription and pioneer factor which functions to specify prostate development [47]. Based on previous experience in genetic manipulation in human ESCs [29], we have inserted the coding sequence of *NKX3-1* into the AAVS1 locus, thereby establishing the AAVS1-iNKX3-1 cell line with inducible overexpression of NKX3-1. This cell line was then directed to differentiate into the UGS cells maintaining high expression of NKX3-1, addressing the issue of NKX3-1 absence during differentiation, which is of great significance in promoting differentiation towards the prostate.

In contrast to previous studies [22–24], our AAVS1-iNKX3-1 cell line provides a flexible resource to study the underlying mechanisms of NKX3-1 to interact with other signals to orchestrate the prostatic lineage and how its expression influences the formation of the final prostate organoids. In addition, we can obtain cells positive for prostatic markers like CK5, CK8/18, P63 prior to 3D culture, a finding not reported in the previous study. Cells co-expressing the aforementioned markers are likely to be in the prostatic precursor stage, while cells specifically expressing these markers have begun to emerge in 3D cultured organoids, aligning with the developmental theory of the prostate [1]. Moreover, we have been able to rapidly establish prostatic organoids in vitro through directed differentiation of human ESCs, independent of co-culturing with the mesenchyme of the urogenital sinus, thereby excluding interference from other types of cells, delving more deeply into the mechanisms underlying the prostatic differentiation and contribute to elucidating the developmental process of the prostate.

Our study has some limitations awaiting to be solved in future. First, the formed organoids lacked complete basal cell epithelium and interstitial structure and we did not observe a sufficient number of basal epithelial cells from organoids, which were far away from the primary mature prostate. For instance, primary prostate organoids possess basal epithelial cells that express high levels of CK5 and P63, as well as luminal epithelial cells with high levels of AR and CK18, these cells secrete PSA protein [23, 24, 48]. The maturation of prostate organoids is a complex process possibly mediated by multiple signaling pathways. Dissection of the microenvironment of prostate or analyzing the mesenchymal-epithelial interaction between UGM and UGE might provide additional signals, which could facilitate to improve the media for maturation. Second, our strategy requires the gene reprogramming to overexpress *NKX3-1*, which might differ from the endogenous activation of *NKX3-1* during differentiation. Studies have shown that AR can activate the transcription of *NKX3-1* in the luminal and stromal cells of the prostate [49], indicating matured structure might facilitate the endogenous expression of NKX3-1. Additionally, the efficiency of exogenous NKX3-1 expression induced by Doxy is only about 20% at UGS stage relative to about 100% at ESCs, indicating that more complicated mechanism during differentiation mediated the silence of NKX3-1 even in the safe harbor site. Considering that many additional signals, such as FGF, HOX, SHH, and BMP, also play a role in the development of the prostate [50], we will further explore more effective factors promoting the expression of NKX3-1 and driving the differentiation towards the prostate direction.

Nevertheless, we have constructed the prostate organoids from human ESCs, which might be applied in basic research and translational studies, such as evaluating the toxicity of relevant drugs on human prostate development and function. For instance, a previous study utilized prostate organoids and found Bisphenol-A at lower doses indeed enhanced the growth and branching of the prostate, whereas higher exposures drove apoptosis and restricted the growth of the prostate [48]. In addition, our differentiation protocol mimicked the major developmental events, which provides an alternative model to identify genes and signaling pathways associated with prostate development [24]. Furthermore, well-established prostate organoids can be utilized for disease modeling, organ repair, and biomarker discovery, offering a broad spectrum of potential applications [51].

## Conclusion

In summary, we have developed an in vitro differentiation protocol to induce human ESCs into to UGS and prostatic lineage cells. In addition, we have constructed a human ESC line overexpressing *NKX3-1* in endogenous AAVS1 site. Eventually, we obtained prostatic cells positive for AR, FOXA1, NKX3-1, CK5, CK8/18 and P63, which could further form organoids with characteristics resembling the native prostate by a Matrigel-based 3D culture system. Therefore, this study provides useful strategy and resource to construct human prostate-like cells and organoids, which should facilitate the understanding on developmental process of human prostate and the pathophysiology mechanism of prostate-related diseases.

### Electronic supplementary material

Below is the link to the electronic supplementary material.

Supplementary Material 1

## Data Availability

All data analyzed in this study are included in this published article.

## Abbreviations

AR: Androgen receptor
ARE: Androgen response element
DE: Definitive endoderm
DHT: Dihydrotestosterone
Doxy: Doxycycline
ECM: Extracellular matrix
ESC: Embryonic stem cell
HG: Hindgut
iPSC: Induced pluripotent stem cell
KGF: Keratinocyte growth factor
PSC: Pluripotent stem cell
RA: Retinoic acid
SVM: Spermatic vesical mesenchyme
UGS: Urogenital sinus
UGE: Urogenital sinus epithelium
UGM: Urogenital sinus mesenchyme
