# Supplementary material for: Prostatic lineage differentiation from human embryonic stem cells through inducible expression of NKX3-1
Source: Stem Cell Res Ther. 2024 Sep 2;15:274. doi: 10.1186/s13287-024-03886-y (PMC11367998; doi:10.1186/s13287-024-03886-y)
Supplement: Supplementary file 1 — Supplementary Material 1 [file 13287_2024_3886_MOESM1_ESM.pdf]

Supplemental Information

Prostatic lineage differentiation from human embryonic stem cells through inducible expression of NKX3-1

Supplementary figures and legends, S1-S4

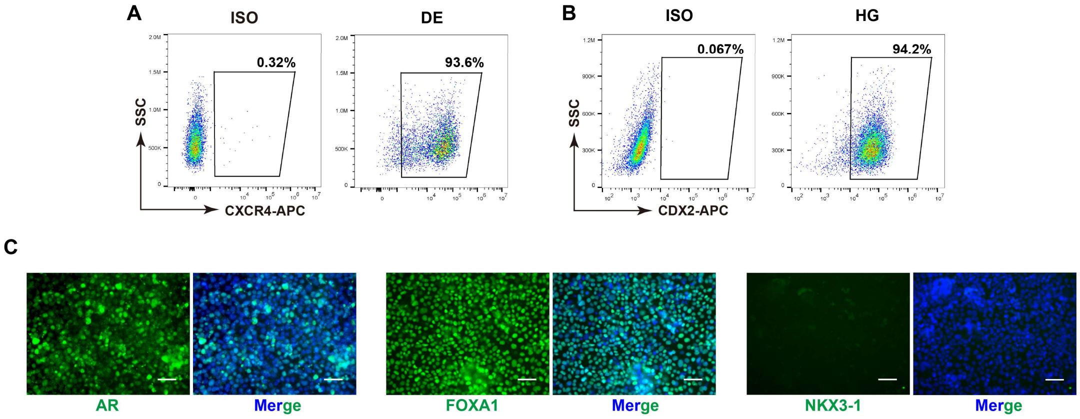

Figure S1. UGS differentiation of H9 through DE and hindgut stages. (A-B) Flow cytometric results of CXCR4 at DE stage (A) and CDX2 at HG stage (B) (DE: definitive endoderm cell, HG: hindgut cell, ISO: isotype control). (C) Immunofluorescence results of AR, FOXA1 and NKX3-1 at UGS stage. Scale bar = 50 μm.

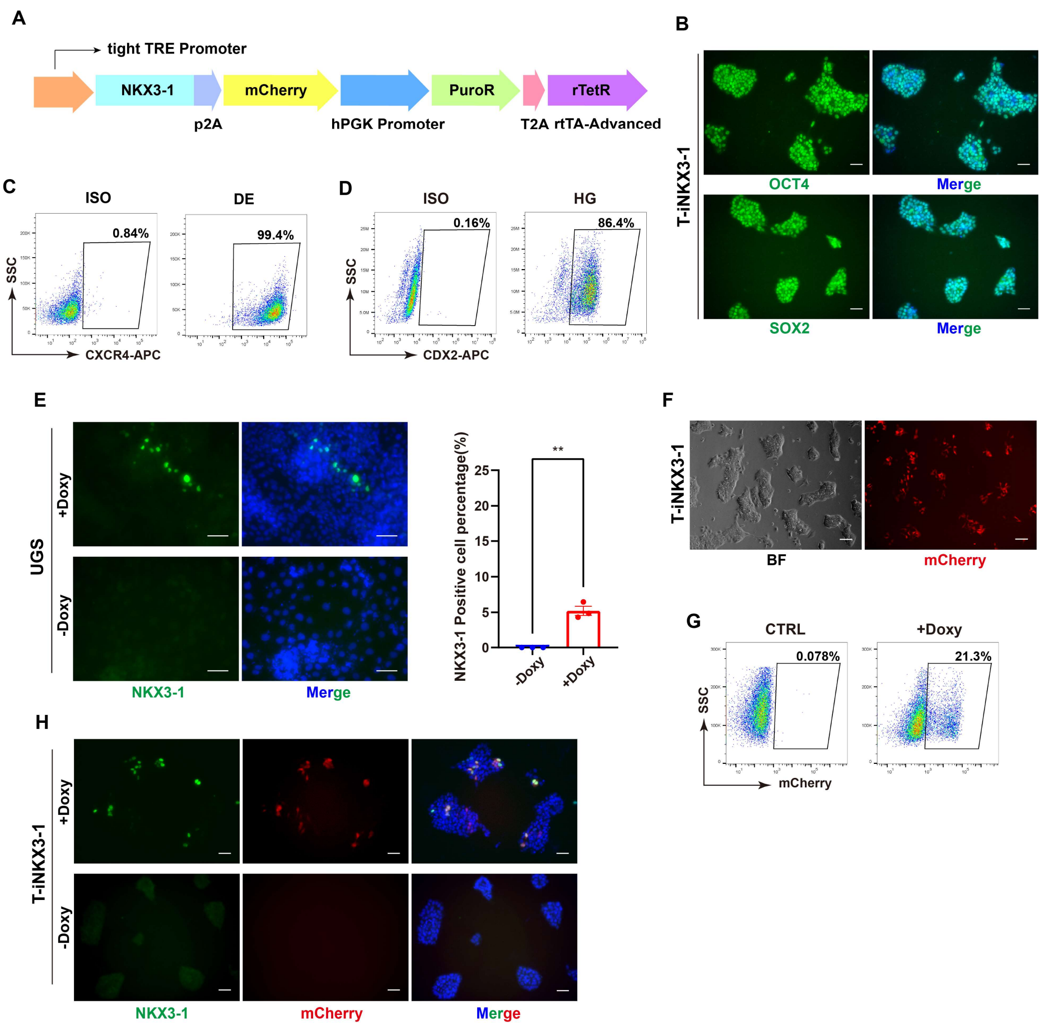

Figure S2. Construction and characterization of transgenic NKX3-1 expression ESC line T-iNKX3-1. (A) Schematic diagram of the construction of the mCherry-NKX3-1 overexpression vector. (B) Immunofluorescence results of the pluripotency markers OCT4 and SOX2 in T-iNKX3-1 cells. Scale bar = 50 μm. (C) Flow cytometry results of CXCR4-positive cells at DE stage (DE: definitive endoderm cell, ISO: isotype control). (D) Flow cytometry results of CDX2-positive cells at HG stage (HG: hindgut cell). (E) Immunofluorescence results of NKX3-1 in the cells of the urogenital sinus, with the experimental group being treated with Doxy induction and the control group not receiving any induction, during the process of hindgut differentiation towards the urogenital sinus and statistical results of NKX3-1-positive cells (n=3) (UGS: urogenital sinus cell, Doxy: Doxycycline). Scale bar = 50 μm. (F) Brightfield and mCherry immunofluorescence results of T-iNKX3-1, with a scale bar of 50 μm (BF: brightfield). (G) Flow cytometry results of mCherry-positive cells after 48 hours of Doxy induction in T-iNKX3-1 (CTRL: control). (H) Immunofluorescence results of NKX3-1 and mCherry after 48 hours of Doxy induction in T-iNKX3-1, with the control group not receiving Doxy induction. Scale bar = 50 μm. ** $p < 0.01$.

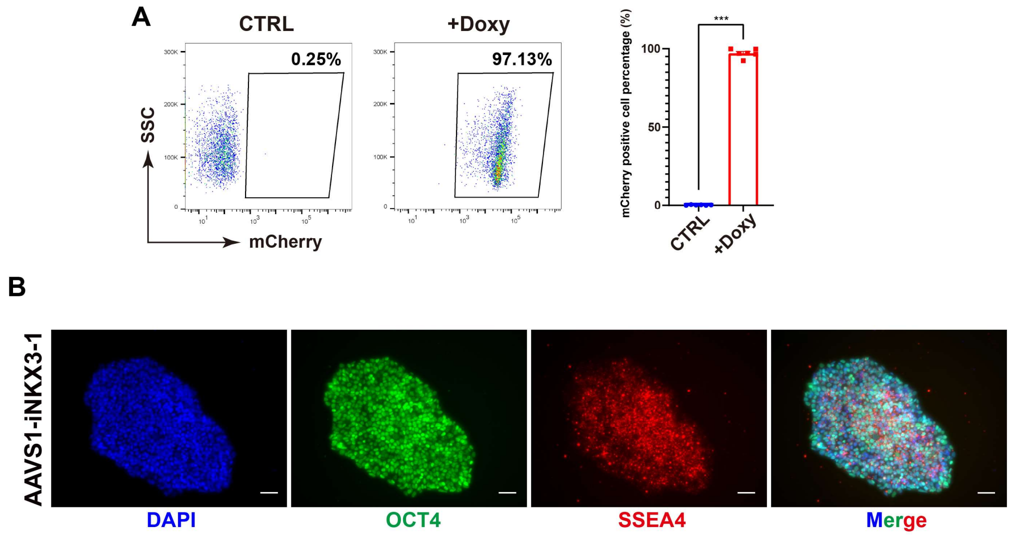

Figure S3. Characterization of AAVS1-iNKX3-1 cell line. (A) Flow cytometry results of mCherry after 48 hours of induction with Doxy (CTRL: control, Doxy: Doxycycline), along with the quantitative analysis (n=6). (B) The immunofluorescence results for OCT4 and SSEA4. Scale bar = 50 μm. *** $p < 0.001$.

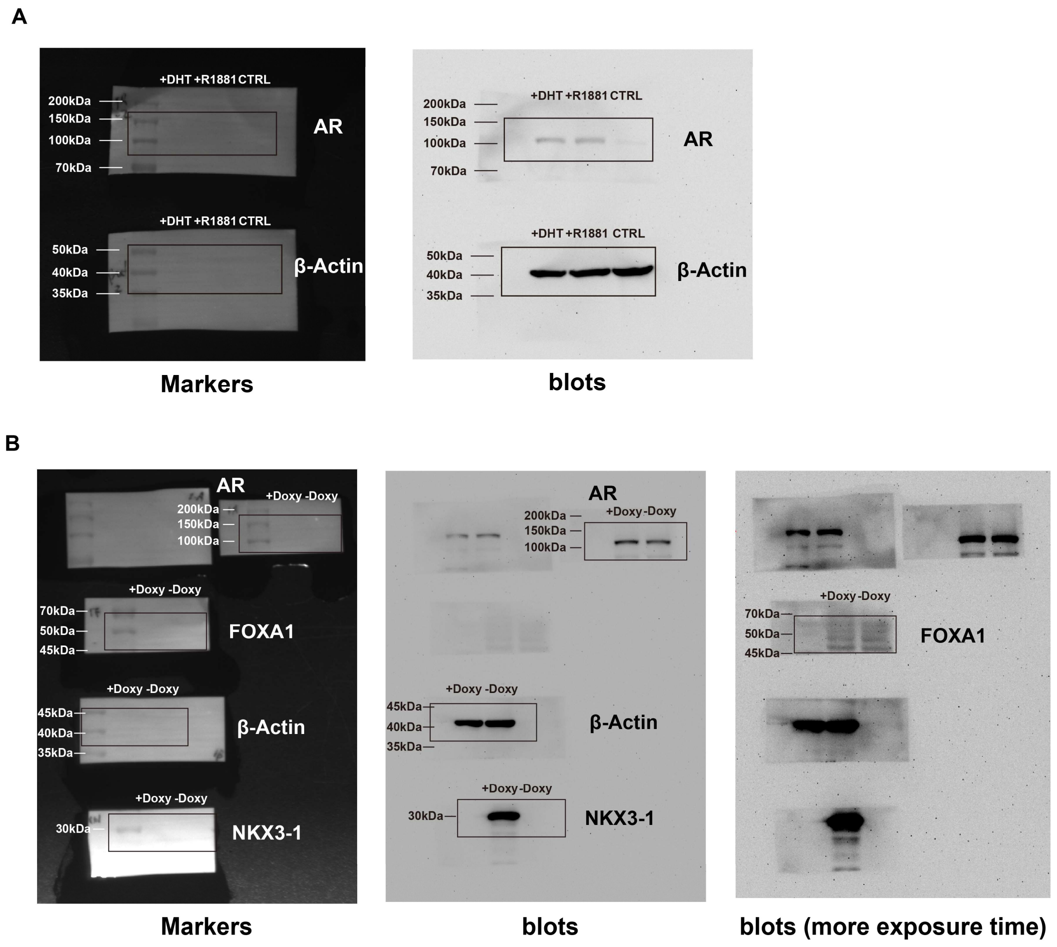

Figure S4. Uncropped full-length blots/gels. (A) Markers and blots images of Fig. 4B (CTRL: control). (B) Markers and blots images of Fig. 4D, gels with markers are shown left.
